# Supplementary material for: Changes in Striatal Medium Spiny Neuron Morphology Resulting from Dopamine Depletion Are Reversible
Source: Cells. 2020 Nov 9;9(11):2441. doi: 10.3390/cells9112441 (PMC7695336; doi:10.3390/cells9112441)
Supplement: Supplementary file 1 [file cells-09-02441-s001.pdf]

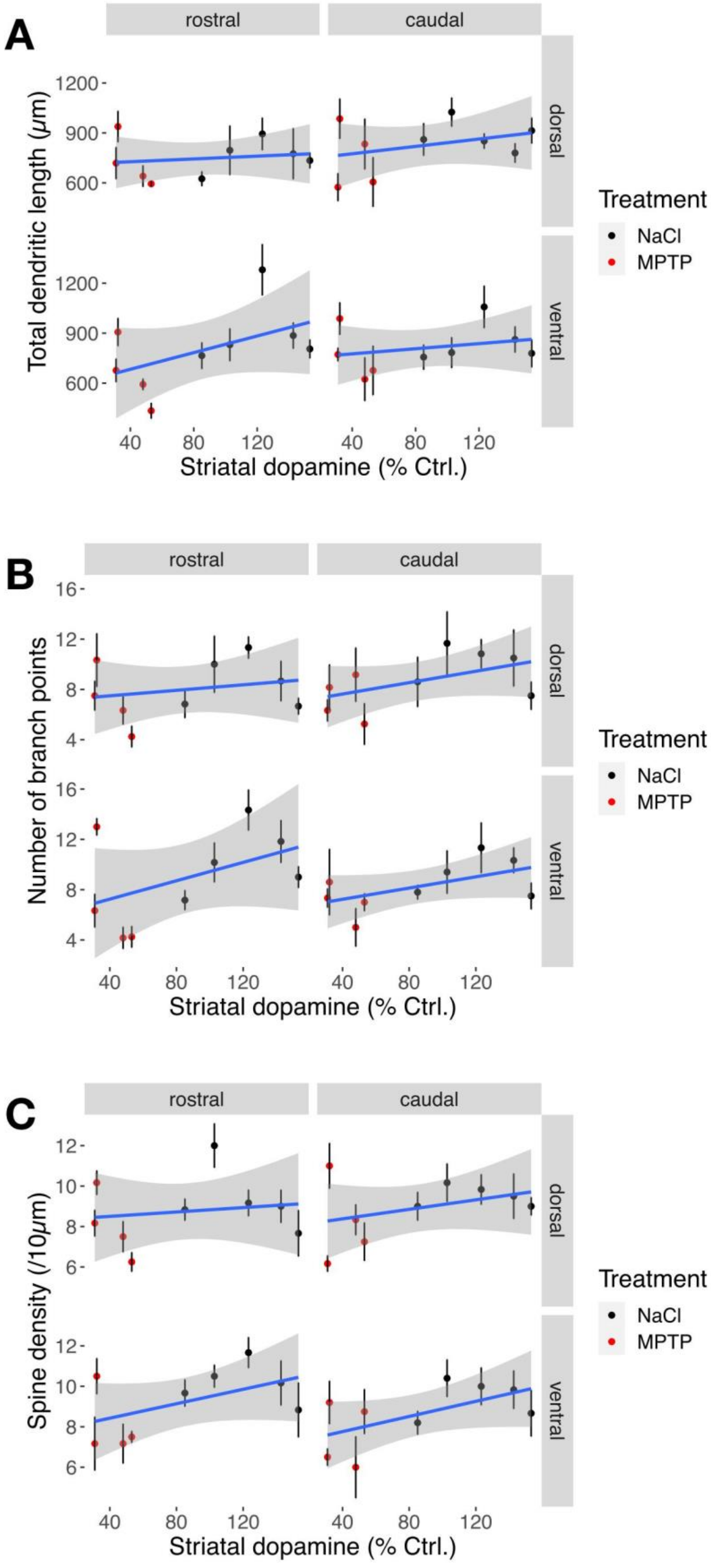

supplemental Figure S1

Supplemental Figure S1: Per-animal analysis of MSN morphology 21 days after MPTP

For this display, values for total dendritic length (A), number of dendritic branch points (B) and spine density (C) were summarized for each animal and displayed against the concentration of striatal dopamine in that animal. Black dots represent animals with NaCl administration, red dots represent animals with MPTP administration. MSN were divided into four regions based on their location in the striatum (rostral or caudal slices, dorsal or ventral location in the slice). Error bars are the SEM from all MSN in the group represented by the dot. As explained in the text, the correlation of MSN morphology parameters is driven more strongly by the NaCl vs MPTP group than by the dopamine concentration, indicating for instance that MSN morphology is not affected by differences in baseline dopamine concentration in untreated animals. In addition, the variance in dopamine concentrations might result from the measurements. There are no systematic differences between striatal subregions with the possible exception that the modulation by dopamine seems to be most pronounced in ventral and rostral striatum. As detailed in the text, the correlation with dopamine concentration was significant, but the differences between subregions was not. A more detailed analysis of regional differences of MSN adaptation would require knowledge of regional differences in the density of dopaminergic axon terminals, which is not available for this dataset.
